# Case Report: Fenestration-free non-obstructive left atrial membrane overlapping with the cor triatriatum spectrum: multimodality imaging and three-year follow-up

**DOI:** 10.3389/fcvm.2026.1868310

**Published:** 2026-06-10

**Authors:** Ali Hakan Konuş

**Affiliations:** Department of Cardiology, Bingöl State Hospital, Bingöl, Türkiye

**Keywords:** cardiac computed tomography, congenital atrial anomaly, cor triatriatum spectrum, left atrial membrane, multimodality imaging, transesophageal echocardiography

## Abstract

Non-obstructive left atrial membranous anomalies may overlap anatomically with the cor triatriatum spectrum while lacking the classical hemodynamic features of functional left atrial compartmentalization. We report a 23-year-old woman referred for echocardiographic evaluation, in whom a left atrial membrane was characterized by multimodality imaging. Transesophageal echocardiography demonstrated a membrane originating adjacent to the left atrial appendage ostium and attaching to the interatrial septum at the aortic rim, without detectable transmembranous flow. Mitral inflow and pulmonary venous Doppler patterns were normal, and left atrial appendage emptying velocity was preserved (58 cm/s). Contrast-enhanced cardiac computed tomography confirmed the membrane's anatomical configuration, its spatial relationship to the left atrial appendage and interatrial septum, and the superior localization of pulmonary venous inflow relative to the membrane. Conservative management was adopted, and three-year follow-up remained entirely event-free, with no arrhythmia, thromboembolic event, or hemodynamic progression. This case illustrates that apparent anatomical continuity does not necessarily imply physiological obstruction in left atrial membranous anomalies and underscores the role of integrated multimodality assessment in guiding long-term management.

## Introduction

Left atrial membranous structures represent an uncommon spectrum of congenital anomalies, most frequently described in the context of cor triatriatum sinister (1, 2). Classical forms are characterized by a fibromuscular membrane dividing the left atrium into two chambers, with fenestrations determining the degree of pulmonary venous and mitral inflow obstruction. However, apparent anatomical continuity of a membrane may not necessarily imply functional obstruction.

With the expanding use of advanced cardiac imaging, atypical and incomplete left atrial membrane variants are increasingly identified, including in asymptomatic adults (3). Accurate anatomical and functional assessment is essential to distinguish true membranous structures from supramitral ring, normal atrial ridges, or imaging artifacts, and to guide appropriate long-term management in adults with congenital heart disease (4).

## Case presentation

A 23-year-old woman with no prior medical history was referred for echocardiographic evaluation because of intermittent chest pain. Physical examination and laboratory tests were unremarkable. Electrocardiography showed no ischemic changes, and cardiac biomarkers were within normal limits, making an acute coronary syndrome unlikely. Given the atypical characteristics of the chest pain and the unremarkable initial evaluation, no further coronary assessment was pursued. Transthoracic echocardiography revealed a linear echogenic membranous structure within the left atrium, clearly distinct from the mitral valve apparatus (Figure 1). No transmitral gradient was detected. Transesophageal echocardiography provided detailed anatomical characterization (Figure 2). At 15°, the membrane originated adjacent to the left atrial appendage ostium and extended anteriorly toward the interatrial septum. At 75°, it attached to the interatrial septum at the aortic rim, corresponding to the septal segment adjacent to the aortic root. Color Doppler imaging showed no transmembranous flow at any imaging plane. Mitral inflow velocities were normal, and pulmonary venous flow patterns showed no evidence of upstream obstruction. Left atrial appendage emptying velocity was preserved (58 cm/s), with no spontaneous echo contrast or thrombus. Contrast-enhanced cardiac computed tomography confirmed a linear ridge-like membranous structure within the left atrium. Coronal reconstruction further supported the anatomical configuration identified on transesophageal echocardiography (Figure 3). On axial images, the right superior, right inferior, and left common pulmonary veins were visualized draining superior to the membrane. Given the absence of obstruction or hemodynamic compromise, conservative management was adopted. Over three years of clinical and echocardiographic follow-up, the patient remained asymptomatic, with no recurrence of the presenting chest pain, no atrial arrhythmia, thromboembolic event, or evidence of echocardiographic progression.

**Figure 1:**
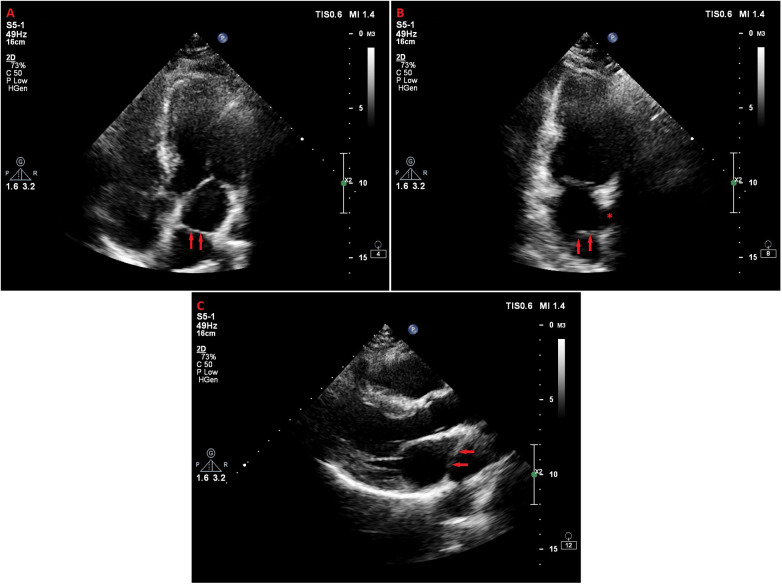
Transthoracic echocardiographic identification of a left atrial membrane. **(A)** Apical five-chamber view demonstrating a thin, linear echogenic structure within the left atrium (arrow), distinct from the mitral valve apparatus. **(B)** Apical two-chamber view showing the left atrial membrane (arrow) extending superiorly. The left atrial appendage is indicated by an asterisk (*). **(C)** Parasternal long-axis view confirming the presence of a linear membranous structure within the left atrium (arrow).

**Figure 2:**
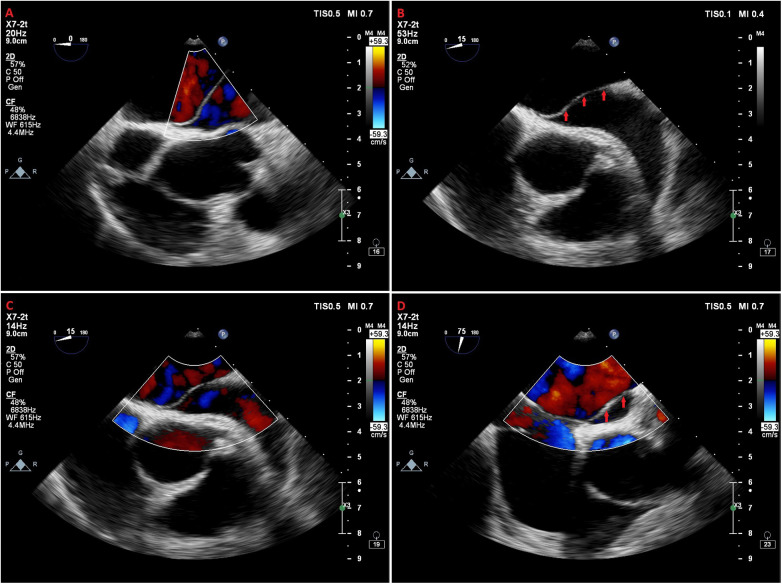
Transesophageal echocardiographic anatomical characterization. **(A)** Mid-esophageal view at 0° with color Doppler showing no detectable transmembranous flow. **(B)** Mid-esophageal view at 15° illustrating the membrane (arrows) originating adjacent to the left atrial appendage ostium. **(C)** Color Doppler at 15° confirming absence of transmembranous flow. **(D)** Mid-esophageal view at 75° demonstrating attachment to the interatrial septum at the aortic rim.

**Figure 3:**
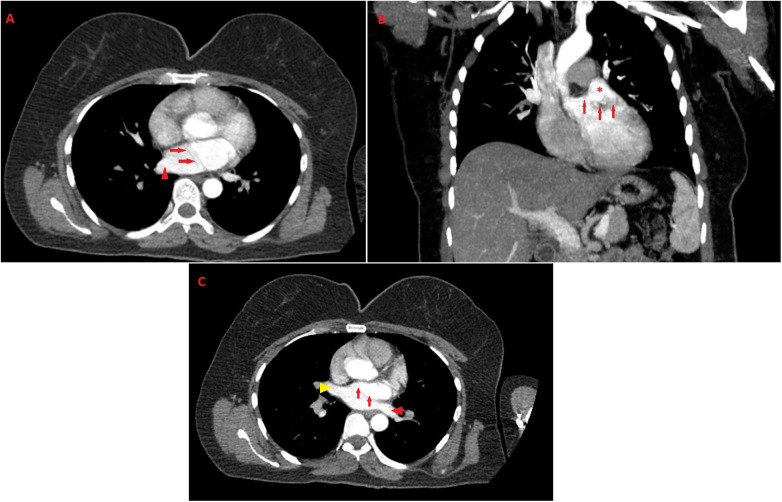
Multiplanar cardiac computed tomography characterization of the left atrial membrane. **(A)** Axial contrast-enhanced image demonstrating a linear ridge-like membranous structure within the left atrium (arrows); the right superior pulmonary vein (arrowhead) is visualized draining superior to the membrane. **(B)** Coronal reconstruction illustrating the membranous structure (arrows) in proximity to the left atrial appendage ostium. The left atrial appendage is indicated by an asterisk (*). **(C)** Axial image demonstrating the right inferior pulmonary vein (yellow arrowhead) and left common pulmonary vein (red arrowhead) draining superior to the membrane, further illustrating the anatomical relationship between pulmonary venous inflow and the membranous structure (arrows).

## Discussion

The present case illustrates a non-obstructive left atrial membranous anomaly sharing anatomical features with the cor triatriatum spectrum without producing true functional atrial partitioning or hemodynamic compromise. The clinical relevance of such structures depends on anatomical location, relationship to the mitral valve apparatus, and the presence of hemodynamic obstruction. The principal differential diagnoses include cor triatriatum sinister, supramitral ring, and anatomical ridges or imaging artifacts.

Although intermittent chest pain was the presenting complaint, its relationship to the left atrial membrane remains uncertain. The membrane's non-obstructive nature, combined with the absence of ischemic electrocardiographic changes, normal cardiac biomarkers, and absence of symptom recurrence during follow-up, makes a direct hemodynamic mechanism unlikely. A non-cardiac etiology cannot be excluded.

Classical cor triatriatum is characterized by division of the left atrium into proximal and distal chambers, with fenestrations determining the degree of pulmonary venous obstruction (1). In the present case, the membrane did not create functionally distinct chambers, had no identifiable fenestration, and was not associated with elevated transmitral gradients or pulmonary venous flow abnormalities, supporting anatomical overlap with the cor triatriatum spectrum despite the absence of functional atrial partitioning (3). Supramitral ring was excluded because the structure was anatomically remote from the mitral annulus, with normal mitral inflow velocities. Consistent visualization across modalities and concordant transesophageal and computed tomographic findings argue against an imaging artifact. The defined septal attachment at the aortic rim and extension toward the interatrial septum further distinguish this structure from typical atrial ridges confined to the appendage region (5, 6).

Left atrial membranous structures may be characterized along two descriptive axes: presence or absence of fenestration, and presence or absence of hemodynamic obstruction, which yields four descriptive categories (Table 1). This framework is intended as a descriptive conceptual aid rather than a validated classification system. Fenestrated obstructive membranes correspond to classical cor triatriatum. Fenestrated non-obstructive variants, often described as incomplete or asymptomatic forms, have also been reported (7). Fenestration-free non-obstructive membranes, as in the present case, represent a less commonly described anatomical and hemodynamic configuration. Isolated reports of left atrial membranes without visible fenestration and without Doppler evidence of obstruction have been described (8). In the report by Lindauer et al. (8), anatomical classification remained uncertain despite computed tomographic confirmation of the membrane, likely reflecting the more limited anatomical characterization available in that case. In contrast, the present case underwent detailed multiplanar transesophageal and computed tomographic assessment, without identifiable transmembranous communication or evidence of hemodynamic obstruction. Taken together, the present case suggests that apparent anatomical continuity does not necessarily imply physiological obstruction. Existing classification systems, including the Loeffler scheme, primarily categorize left atrial membranes according to the degree of fenestration and the resulting hemodynamic effect (1, 2). However, the present case does not conform to any of the three Loeffler groups, as it combines apparent anatomical continuity with the absence of hemodynamic significance, a combination not explicitly addressed by conventional frameworks. In contrast to the predominantly pediatric presentation of obstructive cor triatriatum variants (2), the present case illustrates that fenestration-free non-obstructive membranes may remain clinically silent and undetected until adulthood.

**Table 1:** Descriptive conceptual framework for anatomical and hemodynamic characterization of left atrial membranous structures.

	Obstructive	Non-obstructive
Fenestrated	Classical cor triatriatum	Fenestrated non-obstructive
Fenestration-free	Fenestration-free obstructive	Fenestration-free non-obstructive (present case)

Categories are based on two independent axes: presence or absence of membrane fenestration, and presence or absence of hemodynamic obstruction. The highlighted category represents the anatomical and hemodynamic configuration observed in the present case.

Although absence of fenestration is often presumed to imply obstruction, physiological impact may depend on whether the membrane circumferentially isolates pulmonary venous return from the mitral inflow pathway. In the present case, the integrated anatomical and Doppler findings did not support effective physiological partitioning, despite apparent anatomical continuity of the membrane and superior localization of pulmonary venous inflow on cross-sectional imaging. This configuration distinguishes the present lesion from classical cor triatriatum, in which a circumferential membrane creates a true proximal chamber receiving pulmonary venous return upstream of the mitral valve, with fenestrations determining the degree of obstruction.

Beyond obstruction, theoretical concerns include localized flow stasis or thrombus formation when membranes are situated near the left atrial appendage ostium. In the present case, preserved appendage emptying velocity, absence of spontaneous echo contrast, and event-free long-term follow-up further support the absence of hemodynamic significance of this anatomical configuration. These findings do not support routine anticoagulation in the absence of established risk factors and are consistent with a conservative surveillance approach in adults with congenital heart disease (4). Periodic echocardiographic follow-up to monitor for potential changes in membrane morphology or appendage flow dynamics may be considered in this setting.

## Conclusion

This case illustrates that some non-obstructive left atrial membranous anomalies may share anatomical features with the cor triatriatum spectrum without producing true functional atrial partitioning. The present findings suggest that apparent anatomical continuity does not necessarily imply physiological obstruction, and that conservative long-term management is appropriate in the absence of hemodynamic compromise. The integrated anatomical, Doppler, and follow-up findings supported the absence of hemodynamic significance of this anatomical configuration. Integrated multimodality imaging is essential to guide accurate anatomical characterization and long-term surveillance in adults with left atrial membranous anomalies not fully addressed by existing classification systems.

## Data Availability

The original contributions presented in the study are included in the article/Supplementary Material, further inquiries can be directed to the corresponding author.
